# Supplementary material for: Factors Associated with Levels of Organophosphate Pesticides in Household Dust in Agricultural Communities
Source: Int J Environ Res Public Health. 2022 Jan 13;19(2):862. doi: 10.3390/ijerph19020862 (PMC8775797; doi:10.3390/ijerph19020862)
Supplement: Supplementary file 1 [file ijerph-19-00862-s001.zip › ijerph-1490144-supplementary.pdf]

Supplementary Information

Table S1. Ion transitions and corresponding fragmentor and collision energy voltages for quantification of OPs in household dust

Compound Name	Precursor Ion	Product Ions	Fragmentor	Collision Energy	Polarity
Acephate	184	143/95	50	4/24	Positive
Acephate-D3	187	143/95	50	4/24	Positive
Dimethoate	230	199/171	80	5/10	Positive
Dimethoate-D6	236	205/131	80	4/20	Positive
Oxydementon-methyl	247	169/109	69	8/28	Positive
Malathion	331	285/127	97	0/8	Positive
Malathion-D6	337	127/99	97	8/20	Positive
Dibrom	378.8	127/109	100	8/40	Positive
Diazinon	305	169/153	85	20/20	Positive
Phorate	261	199/75	54	0/8	Positive
Phosmet	318	160/77	70	12/64	Positive
Bensilide	398	158/77	69	20/56	Positive
Tribufos	315	169/57	93	12/24	Positive
Chlorpyrifos	350	198/97	100	20/20	Positive

Table S2a. Model selection process of covariates considered for modeling total OPs in household dust

Variable	Data type	Levels	Univariable model *p*-value>0.25	Near-zero variance	Not selected by best subsets regression	Removed from consensus model
Visit month[b]	Categorical	January[a]	X			X
		June				
Home type	Categorical	House	X			
		Other (mobile home or apartment)[a]				
Number of rooms	Linear				X	
Number of doors	Linear				X	
Number of windows	Linear		X			
Home ownership	Categorical	Own	X			
		Other (rent or owned by employer)[a]				
**Flooring: carpet[b]**	Indicator	Present vs. not present[a]	X			
Flooring: rugs	Indicator	Present vs. not present[a]	X			
**Window coverings: blinds**	Indicator	Present vs. not present[a]	X			
Window coverings: curtains	Indicator	Present vs. not present[a]	X		X	
Window coverings: shades	Indicator	Present vs. not present[a]			X	
Warm-blooded pet(s)	Indicator	Owned vs. not owned[a]			X	
**Heating sources**	Categorical	Clean (only electric sources used)[a]				
		Dirty (gas and/or oil sources used)				
		None (no heating sources used)				
Cooling: AC	Indicator	Used vs. not used[a]	X		X	
Cooling: window units	Indicator	Used vs. not used[a]	X		X	
Cooling: open windows	Indicator	Used vs. not used[a]	X		X	
**Cooling: fans/house fans**	Indicator	Used vs. not used[a]				
Household chemicals to control ants/bugs	Indicator	Used vs. not used[a]			X	
Household chemicals to control mice/rodents	Indicator	Used vs. not used[a]	X			
Household chemicals to control weeds	Indicator	Used vs. not used[a]			X	
Household chemicals to control pests	Indicator	Used vs. not used[a]	X			
Flea/tick treatments for warm-blooded pets	Indicator	Used vs. not used[a]	X			
Number of adults living in the household	Linear		X			
Number of children living in the household	Linear		X			
Household occupancy	Linear				X	
**Secondary occupational exposure to agriculture/pesticides[b]**	Indicator	Exposed vs. not exposed[a]				
Secondary occupational exposure to dust	Indicator	Exposed vs. not exposed[a]	X			
Household SHS exposure	Indicator	Exposed vs. not exposed[a]	X			
**Community[b]**	Categorical	Community #1[a]				
		Community #2				
		Community #3				
		Community #4				

[a]Indicates referent level for indicator or categorical variables.

[b]Four variables (carpet flooring, visit month, secondary occupational exposure to agriculture/pesticides, and community) were considered *a priori* to be potentially important factors associated with household dust OP levels. These were included in the consensus model, regardless of whether they were previously eliminated by the model selection approach.

Bold typeface indicates variables that were ultimately selected for the final model

Table S2b. Model selection process of covariates considered for modeling chlorpyrifos in household dust

Variable	Data type	Levels	Univariable model *p*-value>0.25	Near-zero variance	Not selected by best subsets regression	Removed from consensus model
Visit month[b]	Categorical	January[a]	X			X
		June				
Home type	Categorical	House	X			
		Other (mobile home or apartment)[a]				
**Number of rooms**	Linear					
Number of doors	Linear				X	
Number of windows	Linear		X			
Home ownership	Categorical	Own	X			
		Other (rent or owned by employer)[a]				
**Flooring: carpet[b]**	Indicator	Present vs. not present[a]	X			
Flooring: rugs	Indicator	Present vs. not present[a]			X	
**Window coverings: blinds**	Indicator	Present vs. not present[a]	X			
Window coverings: curtains	Indicator	Present vs. not present[a]	X		X	
**Window coverings: shades**	Indicator	Present vs. not present[a]				
Warm-blooded pet(s)	Indicator	Owned vs. not owned[a]	X			
Heating sources	Categorical	Clean (only electric sources used)[a]	X			
		Dirty (gas and/or oil sources used)				
		None (no heating sources used)				
Cooling: AC	Indicator	Used vs. not used[a]	X			
Cooling: window units	Indicator	Used vs. not used[a]	X			
Cooling: open windows	Indicator	Used vs. not used[a]	X			
Cooling: fans/house fans	Indicator	Used vs. not used[a]	X			
**Household chemicals to control ants/bugs**	Indicator	Used vs. not used[a]				
Household chemicals to control mice/rodents	Indicator	Used vs. not used[a]	X			
Household chemicals to control weeds	Indicator	Used vs. not used[a]			X	
Household chemicals to control pests	Indicator	Used vs. not used[a]	X			
Flea/tick treatments for warm-blooded pets	Indicator	Used vs. not used[a]	X			
Number of adults living in the household	Linear		X			
Number of children living in the household	Linear		X			
Household occupancy	Linear		X			
**Secondary occupational exposure to agriculture/pesticides[b]**	Indicator	Exposed vs. not exposed[a]				
Secondary occupational exposure to dust	Indicator	Exposed vs. not exposed[a]			X	
Household SHS exposure	Indicator	Exposed vs. not exposed[a]	X			
**Community[b]**	Categorical	Community #1[a]				
		Community #2				
		Community #3				
		Community #4				

[a]Indicates referent level for indicator or categorical variables.

[b]Four variables (carpet flooring, visit month, secondary occupational exposure to agriculture/pesticides, and community) were considered *a priori* to be potentially important factors associated with household dust OP levels. These were included in the consensus model, regardless of whether they were previously eliminated by the model selection approach.

Bold typeface indicates variables that were ultimately selected for the final model

Table S2c. Model selection process of covariates considered for modeling malathion in household dust

Variable	Data type	Levels	Univariable model *p*-value>0.25	Near-zero variance	Not selected by best subsets regression	Removed from consensus model
Visit month[b]	Categorical	January[a]	X			X
		June				
**Home type**	Categorical	House				
		Other (mobile home or apartment)[a]				
Number of rooms	Linear		X			
Number of doors	Linear				X	
Number of windows	Linear				X	
Home ownership	Categorical	Own	X			
		Other (rent or owned by employer)[a]				
Flooring: carpet[b]	Indicator	Present vs. not present[a]	X		X	X
Flooring: rugs	Indicator	Present vs. not present[a]			X	
Window coverings: blinds	Indicator	Present vs. not present[a]	X			
Window coverings: curtains	Indicator	Present vs. not present[a]	X			
Window coverings: shades	Indicator	Present vs. not present[a]	X			
Warm-blooded pet(s)	Indicator	Owned vs. not owned[a]			X	
Heating sources	Categorical	Clean (only electric sources used)[a]	X			
		Dirty (gas and/or oil sources used)				
		None (no heating sources used)				
Cooling: AC	Indicator	Used vs. not used[a]	X		X	
**Cooling: window units**	Indicator	Used vs. not used[a]				
Cooling: open windows	Indicator	Used vs. not used[a]	X		X	
**Cooling: fans/house fans**	Indicator	Used vs. not used[a]				
Household chemicals to control ants/bugs	Indicator	Used vs. not used[a]			X	
Household chemicals to control mice/rodents	Indicator	Used vs. not used[a]	X			
Household chemicals to control weeds	Indicator	Used vs. not used[a]			X	
Household chemicals to control pests	Indicator	Used vs. not used[a]			X	
Flea/tick treatments for warm-blooded pets	Indicator	Used vs. not used[a]			X	
Number of adults living in the household	Linear				X	
Number of children living in the household	Linear		X			
Household occupancy	Linear				X	
Secondary occupational exposure to agriculture/pesticides[b]	Indicator	Exposed vs. not exposed[a]	X			X
Secondary occupational exposure to dust	Indicator	Exposed vs. not exposed[a]			X	
Household SHS exposure	Indicator	Exposed vs. not exposed[a]	X			
**Community[b]**	Categorical	Community #1[a]				
		Community #2				
		Community #3				
		Community #4				

[a]Indicates referent level for indicator or categorical variables.

[b]Four variables (carpet flooring, visit month, secondary occupational exposure to agriculture/pesticides, and community) were considered *a priori* to be potentially important factors associated with household dust OP levels. These were included in the consensus model, regardless of whether they were previously eliminated by the model selection approach.

Bold typeface indicates variables that were ultimately selected for the final model

Table S3. Household dust OP concentrations (ppb) by community and Kruskal-Wallis test results

**Exposure variable**	**Community #1** (n=21)[a]	**Community #2** (n=9)	**Community #3** (n=16)	**Community #4** (n=4)	**Kruskal-Wallis test**	
	mean (SD)	mean (SD)	mean (SD)	mean (SD)	χ2	p-value
**Acephate**	**84.5 (158.8)[2]**	**5.6 (8.7)**	**8.3 (12.8)**	**0 (0)**	**9.33**	**0.03**
Bensulide	0 (0)	0 (0)	0 (0)	0 (0)	-	-
**Chlorpyrifos**	**46.9 (45.7)[2]**	**8.2 (10.6)**	**51.6 (96.5)**	**35.0 (40.5)**	**8.31**	**0.03**
Diazinon	1.2 (1.7)	0.8 (1.6)	0.7 (1.4)	2.3 (2.8)	2.62	0.45
Dibrom	0 (0)	0 (0)	0 (0)	0 (0)	-	-
**Dimethoate**	**2.8 (2.3)**	**0 (0)**	**2.0 (2.4)**	**0.9 (1.8)**	**11.63**	**0.009**
**Malathion**	**3.7 (3.5)**	**1.4 (2.2)**	**7.4 (7.1)**	**7.8 (8.5)**	**11.45**	**0.01**
Oxydemeton-methyl	19.7 (70.7)	0 (0)	0 (0)	0 (0)	2.82	0.42
Phorate	0 (0)	0 (0)	0 (0)	0 (0)	-	-
**Phosmet**	**0.3 (1.1)**	**1.2 (1.8)**	**2.1 (3.2)**	**0 (0)**	**7.55**	**0.06**
**Tribufos**	**5.1 (10.7)[1]**	**0.4 (1.2)**	**0 (0)**	**0 (0)**	**19.40**	**0.0002**
**Total OPs**	**171.4 (199.5)[3]**	**17.6 (23.9)**	**72.1 (94.2)**	**46.0 (33.3)**	**12.79**	**0.005**

[a]For certain individual OPs, concentrations could not be calculated for some Community #1 samples. Values in this common that do not have a numerical footnote are the mean concentration of OP levels from all 21 samples that were collected from Community #1.
[1]The mean concentration is the average of OP levels from 20 samples.
[2]The mean concentration is the average of OP levels from 19 samples.
[3]The mean concentration is the average of OP levels from 18 samples.
Bold typeface indicates variables that were significant as determined by a 95% CI that did not include the null.

Table S4. Sensitivity analysis models, Community #1 samples only

Variable	Estimate	95% CI	
		Lower	Upper
Total OPs (n=18)			
**Window coverings: blinds[b]**	**-1.32**	**-2.45**	**-0.19**
Heating			
Clean (only electric sources used)[a]	-	-	-
Dirty (gas and/or oil sources used)	-1.16	-2.54	0.22
None (no heating sources used)	-0.88	-2.02	0.25
**Cooling: fans/house fans[b]**	**2.40**	**0.42**	**4.39**
Secondary occupational exposure to agriculture/pesticides[b]	0.25	-1.40	1.89
Flooring: carpet[b]	0.25	-1.02	1.52
Chlorpyrifos (n=19)			
Window coverings: blinds[b]	-1.46	-3.21	0.29
Window coverings: shades[b]	1.94	-1.84	5.72
Number of rooms[b]	-0.63	-1.95	0.69
Secondary occupational exposure to agriculture/pesticides[b]	-1.30	-4.28	1.69
Flooring: carpet[b]	-0.73	-2.87	1.41
Malathion (n=21)			
Home type			
House	0.35	-0.32	1.02
Other (mobile home or apartment)[a]	-	-	-
Cooling: window units[b]	0.48	-0.14	1.10
Cooling: fans/house fans[b]	0.61	-0.42	1.64

[a]Indicates referent level for indicator or categorical variables.

[b]For indicator variables, the referent is the absence of the covariate/exposure in the home (e.g., the referent for \`window coverings: blinds\` is not having blinds in the home).

Bold typeface indicates variables that were significant as determined by a 95% CI that did not include the null.
